# Microalgal Metallothioneins and Phytochelatins and Their Potential Use in Bioremediation

**DOI:** 10.3389/fmicb.2020.00517

**Published:** 2020-04-28

**Authors:** Sergio Balzano, Angela Sardo, Martina Blasio, Tamara Bou Chahine, Filippo Dell’Anno, Clementina Sansone, Christophe Brunet

**Affiliations:** ^1^Stazione Zoologica Anton Dohrn Napoli (SZN), Naples, Italy; ^2^NIOZ Royal Netherlands Institute for Sea Research, Den Burg, Netherlands

**Keywords:** heavy metals, metallothioneins, phytochelatins, microalgal biotechnologies, phycoremediation, cysteine, glutathione, metal-binding proteins

## Abstract

The persistence of heavy metals (HMs) in the environment causes adverse effects to all living organisms; HMs accumulate along the food chain affecting different levels of biological organizations, from cells to tissues. HMs enter cells through transporter proteins and can bind to enzymes and nucleic acids interfering with their functioning. Strategies used by microalgae to minimize HM toxicity include the biosynthesis of metal-binding peptides that chelate metal cations inhibiting their activity. Metal-binding peptides include genetically encoded metallothioneins (MTs) and enzymatically produced phytochelatins (PCs). A number of techniques, including genetic engineering, focus on increasing the biosynthesis of MTs and PCs in microalgae. The present review reports the current knowledge on microalgal MTs and PCs and describes the state of art of their use for HM bioremediation and other putative biotechnological applications, also emphasizing on techniques aimed at increasing the cellular concentrations of MTs and PCs. In spite of the broad metabolic and chemical diversity of microalgae that are currently receiving increasing attention by biotechnological research, knowledge on MTs and PCs from these organisms is still limited to date.

## Introduction

The ability of some microbes to thrive in heavy metal-polluted environments is attracting the interest of the biotechnological industry. Eukaryotic microalgae appear to be particularly suitable to use in bioremediation since they require only sunlight and inorganic nutrients for their growth, can achieve fast growth rates, and are able to compartmentalize heavy metals (HMs) within specific organelles. Biotechnological strategies to improve the removal of HMs from polluted waters made significant progress, although large-scale applications of microalgae-based HM-remediation are still not feasible (Kumar et al., 2015). Two main naturally occurring metal-removal processes are currently under investigation in biotechnological research: passive sorption onto algal biomass (de-Bashan and Bashan, 2010) and active sequestration and transport by specific ligands (Monteiro et al., 2011).

Eukaryotic microalgae are present in six different supergroups (Archaeplastida, Hacrobia, Rhizaria, Excavata, Alveolata, and Heterokontophyta), resulting far more genetically diverse than either terrestrial plants (Archaeplastida), or fungi and animals (Opisthokonta). This leads to a greater diversity in primary and secondary metabolites including organic ligands and metal-binding proteins for microalgae. Since most studies focus on plants, animals, yeasts, and bacteria, a significant proportion of microalgal metabolites are unknown or uncharacterized to date. Many organic ligands and metal-binding proteins from microalgae are thus likely to be unknown, and some of them might reveal useful for phytoremediation or other biotechnological applications. The far greater genetic, enzymatic, and chemical diversity found in microalgae, compared to terrestrial plants, animals, or fungi (Keeling, 2013), coupled with the ability of microalgae to grow with sunlight and inorganic nutrients, makes photosynthetic microorganisms the best candidates for biotechnological applications and HM remediation. Furthermore, the presence of organelles in which HMs can be enclosed, limiting their detrimental effects to cellular metabolism, makes microbial eukaryotes more suitable for HM remediation compared to photosynthetic bacteria. Massive sequencing of microalgal genomes and transcriptomes (Keeling et al., 2014; Blaby-Haas and Merchant, 2019), carried out in the last decade, provided scientists with a plethora of information available on potential proteins and biosynthetic pathways involved in HM detoxification. This includes both polypeptides directly chelating HMs, such as metallothioneins (MTs) and phytochelatins (PCs), as well as compounds biosynthesized to decrease the oxidative stress induced by HMs (Cobbett and Goldsbrough, 2002).

The structure and function of both MTs and PCs in terrestrial plants, along with mechanisms for HM sequestration and compartmentalization, have been described in detail by Cobbett and Goldsbrough (2002). MTs are currently classified in 15 evolutionarily unrelated families (Capdevila and Atrian, 2011), and most information on phylogeny and structure of metal-binding domains has been elucidated in deep detail for plant MTs (Leszczyszyn et al., 2013). Structure, biosynthetic pathways, and activity regulation of PCs and their precursors, which can also be involved in HM sequestration, have been extensively reviewed (Hirata et al., 2005; Mendoza-Cozatl et al., 2005; Pal and Rai, 2010; Noctor et al., 2012; Filiz et al., 2019). The production of PCs by the different marine microalgae (Kawakami et al., 2006) as well as the role of microalgal PCs in HM detoxification mechanisms have also been elucidated (Perales-Vela et al., 2006), whereas fewer studies focused instead on microbial eukaryotes. Ziller and Fraissinet-Tachet (2018) highlighted the distribution and diversity of MTs across the three domains of life. To date, MTs have been identified in a small fraction of microbial eukaryotes, and detailed studies are available for ciliates only (Gutiérrez et al., 2009, 2015). Here, current knowledge on microalgal metal-binding proteins is described, and the state of art of the application of metal-binding proteins in biotechnological applications, with special attention devoted to phycoremediation, is discussed.

## Interactions Between Microalgae and Heavy Metals

Heavy metals are defined as those elements with an atomic number greater than 20 and a density greater than 5 g cm^–3^. They comprise 67 out of 118 chemical elements (Ali and Khan, 2018). Iron, zinc, manganese, nickel, copper, cobalt, and molybdenum are essential for cellular metabolism and are part of the so-called metalloproteins, playing an active role in several cellular functions including electron transport and cellular protection against reactive oxygen species (Chadd et al., 1996; Twining and Baines, 2013). In addition, cadmium can also be considered as an essential metal for some microalgae, since it can replace zinc as a catalyst of carbon anhydrase, as found in *Thalassiosira weissflogii* (Lane and Morel, 2000; Xu et al., 2008).

Uptake of metals into living cells typically occurs in two steps: metal adsorption and transport across the cell membrane, with the latter step usually considered to be rate limiting (Levy et al., 2008). During the first stage, which is metabolism independent, the metal ions are adsorbed onto the cell wall through interaction with functional groups, such as polysaccharides and proteins (Das et al., 2008). After adsorption onto the cell wall, metals can penetrate the cell membrane binding to either ion carriers or low molecular weight thiols, such as cysteine, through an active transport process (Narula et al., 2015). The intracellular concentration of HMs ranges from nanomolar to femtomolar (Bruland and Lohan, 2003), and interspecies differences in metal stoichiometry can occur (Loscher, 1999; Twining et al., 2012).

High HM concentrations can inhibit the activity of biologically important molecules, such as enzymes and transport systems, inactivating their functional groups. In addition, HMs can replace essential metal ions in biomolecules and can trigger the over-production of radical oxygen species (ROS) that can, in turn, damage essential biomolecules (Jalmi et al., 2018). Indeed, all HMs are toxic at high concentrations, and their toxicity can vary even within the same taxonomic group. For example, *Phaeodactylum tricornutum* stopped growing at copper(II) concentrations >1.5 μM (Levy et al., 2008), while another diatom, *Thalassiosira pseudonana* is able to grow at copper and cadmium concentrations as high as 3 and 10 μM, respectively (Biswas and Bandyopadhyay, 2017). Some Chlorophyta appear to tolerate even higher copper concentrations, since *Tetraselmis* sp. and *Dunaliella tertiolecta* can grow with 15 μM of dissolved copper (Levy et al., 2008).

Microalgal cells need to avoid both micronutrient deficiency and micronutrient toxicity and thus need to incorporate metals from their surrounding environment at optimal concentrations. A range of mechanisms is applied to maintain optimal concentrations of HMs, such mechanisms include organic ligand formation and exudation, changes in membrane permeability, induction of stress proteins or antioxidants, release of metals into solution, or binding of metals at non-metabolically active sites (Luoma and Rainbow, 2005).

A wide range of organic ligands, also known as transporters, mediates the transport of excess HMs across the cytosol toward chloroplasts, mitochondria, and vacuoles (Cobbett and Goldsbrough, 2002; Mendoza-Cozatl et al., 2002; Aviles et al., 2003; Soldo et al., 2005; Blaby-Haas and Merchant, 2012). Several proteins, known as Group-A transporters, are present across both the plasma and vacuole membranes and allow the transport of metals from the outer environments and the vacuole, respectively, toward the cytoplasm. Group-B transporters are instead present across mitochondrial and vacuolar membranes and are responsible for decreasing the cytoplasmic concentration of metals (Blaby-Haas and Merchant, 2012). Furthermore, microorganisms can produce other organic ligands, such as porphyrins and siderophores, that are known to chelate iron (Hutchins et al., 1999). Also, the complexation of HMs to organic ligands lowers the toxicity of HMs, and a range of ligands can be released by phytoplankton. Copper(II) supplied to cultures of *D. tertiolecta* was found to be complexed by algal exudates or adsorbed onto cell surfaces (Gonzalez-Davila et al., 1995). It has been suggested that at least 99.9% of copper in seawater is complexed by strong organic ligands and that copper toxicity in the water column would be much higher than that observed without organic complexation (Biswas and Bandyopadhyay, 2017). Organic acids can also chelate metals, as shown in higher plants. For example, Sun et al. (2011) found a positive correlation between both tartaric and malic acid and cadmium concentration within the leaves of *Rorippa globosa*. Ueno et al. (2005) identified a cadmium–malate complex within the leaves of *Thlaspi caerulescens* and suggested that such complexation mostly occur in the vacuoles. Cadmium complexation by citric, malonic, and malic acids was also suggested to occur in the cellular vacuole of *Dittrichia viscosa* (Fernández et al., 2014).

Phytochelatins and MTs are two polypeptides mostly involved in metal binding, and a crucial role is played by the sulfide groups of the cysteine residues present in these polypeptides. Metal cations exhibit indeed a chemical affinity toward the partial negative charge associated with the sulfide groups of cysteine, resulting in the formation of HM–MT and HM–PC complexes. Both MTs and PCs can, thus, scavenge HMs minimizing their detrimental effects within the cell. Peptides or amino acids that do not contain sulfide groups can also play a role in metal binding. For example, *Tetraselmis tetrathele* was found to release a cysteine-free peptide (arg–arg–glu) for mercury scavenging (Satoh et al., 1999).

## Metallothioneins in the Microalgal Realm

**FIGURE 1:**
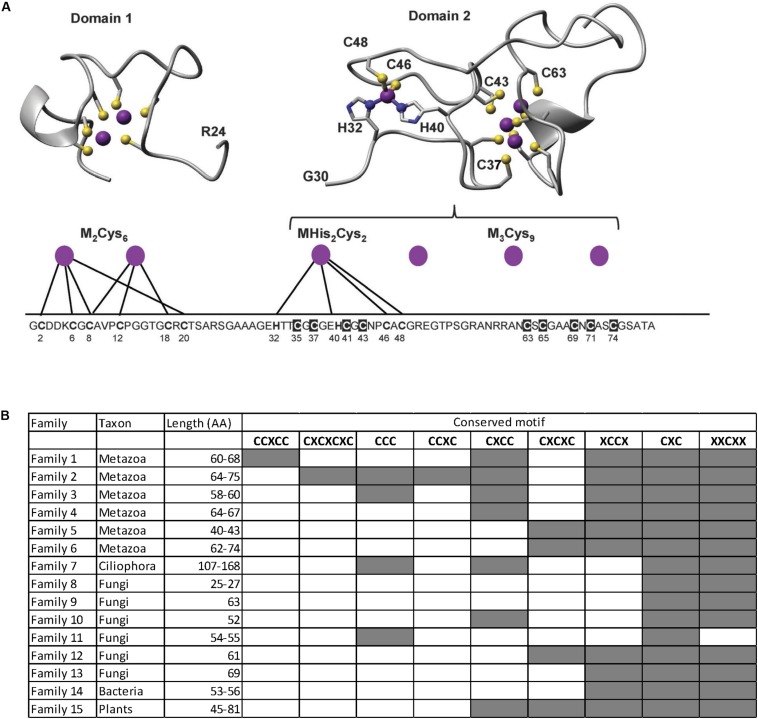
**(A)** Tertiary structure and active sites for metal binding in Type 4 plant metallothioneins from wheat. Adapted from Figure 9 in Leszczyszyn et al. (2013). **(B)** Occurrence of cysteine-rich motifs in the different metallothionein (MT) families described to date. Adapted from Figure 1D in Ziller et al. (2017). License to publish these figures has been obtained from the original publishers, the Royal Society of Chemistry (Metallomics) and Elsevier (Journal of Inorganic Biochemistry).

Metallothioneins are ubiquitous in living organisms and play important roles for both the supply of essential metals to the cell and the transport of toxic metals into other organelles (Capdevila and Atrian, 2011). They have also been suggested to act as free-radical scavengers, thus protecting cells from oxidative stress (Thornalley and Vasak, 1985). MTs are located in the cytosol and are typically defined as small (≤300 amino acids) proteins containing few (<10%) aromatic residues and a high proportion (15–35%) of cysteine (Ziller and Fraissinet-Tachet, 2018), which coordinate metal cations. The length and cysteine content of MTs vary among living organisms; for example, MTs from ciliates are longer than those from other microbial eukaryotes (Gutiérrez et al., 2011), and MTs from Metazoa typically exhibit a greater cysteine content (Ziller and Fraissinet-Tachet, 2018). MTs from plants have been characterized in more detail compared to MTs from other taxa. They have been grouped in four types, and both tertiary structure and metal-binding mechanisms have been elucidated for type 4 plant MTs (Leszczyszyn et al., 2013). Cysteine and, to a lesser extent, histidine residues play a crucial role in metal binding (Leszczyszyn et al., 2010). Each metal cation can be coordinated by several cysteine units (Figure 1A). Type 4 plant MTs consist of a short N-terminal metal-binding domain with a cysteine-rich region and a longer C-terminal domain containing two cysteine-rich regions (Figure 1A). The secondary and tertiary structure of MTs change after HM binding (Leszczyszyn et al., 2013), and HM–MT complexes can vary in shape according to the number of bound cations (Bell and Vallee, 2009). MTs are difficult to classify based on their 3D structure because MT folding varies according to the number and type of coordinated cations.

MTs consist of at least 15 distinct families resulting from convergent evolution as evidenced by the very low sequence similarity occurring among them (Capdevila and Atrian, 2011). Each MT family includes evolutionarily related proteins from organisms belonging to the same taxonomic group. Nine different cysteine-rich conserved motifs have been found across the different MT families (Figure 1B): XXCXX, CXC, XCCX, CXCXC, CXCC, CCXC, CCC, CXCXCXC, and CCXCC. Each MT family contains one or more family-specific motifs (Ziller and Fraissinet-Tachet, 2018). To date, the majority of known eukaryotic MTs have been identified in fungi, Metazoa, plants, and ciliates, whereas very few studies focused on other eukaryotic classes (Ziller and Fraissinet-Tachet, 2018). Based on the arrangements of cysteine in cysteine-rich motifs, Capdevila and Atrian (2011) classified MTs into six families that include one family of proteins from Metazoa, six families from fungi, one from ciliates, one from bacteria, and one from plants (Figure 1B). Since MTs from different families are phylogenetically unrelated, they exhibit a huge variability in length and cysteine-rich motifs (Figure 1B). High variability in the amino acid sequence of MTs can be observed even within the same family of evolutionarily related proteins. For example, Leszczyszyn et al. (2013) reported a broad MT heterogeneity within plants (Family 15), and plant MTs were classified into four types, based on the number and position of the different cysteine-rich motifs within their primary amino acid sequence (Leszczyszyn et al., 2013). Yet, metatranscriptomic analysis of soil microbiome revealed the presence of new cysteine-rich proteins that are involved in cadmium and zinc tolerance (Lehembre et al., 2013), suggesting that the diversity of MTs in nature is much wider than that currently known with more MT families likely to occur.

**FIGURE 2:**
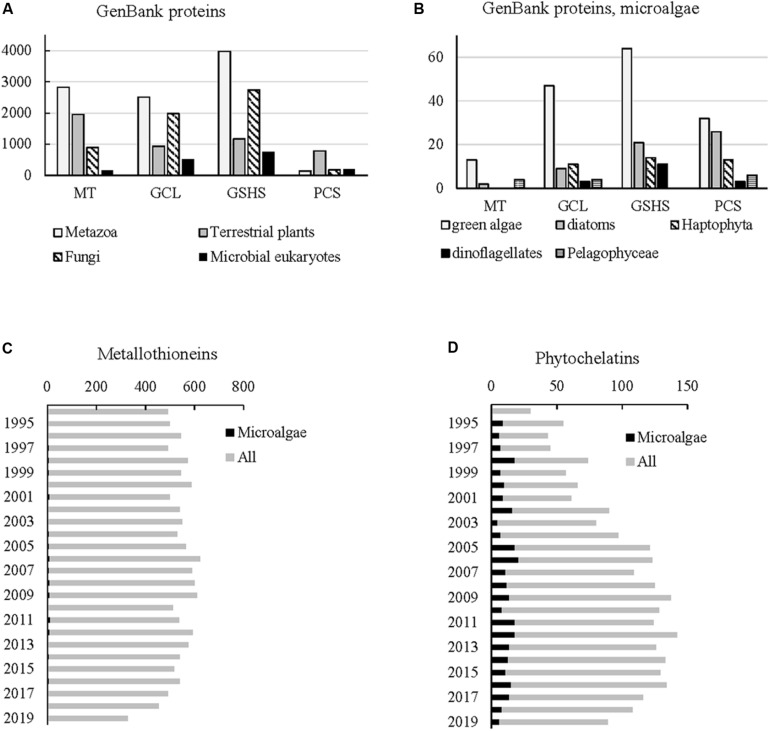
Current knowledge about metallothioneins and phytochelatins. **(A,B)** Taxonomic origin of the GenBank proteins annotated as metallothionein (MT), glutamate–cysteine ligase (GCL), glutathione synthase (GSH), and phytochelatin synthase (PCS) for **(A)** all eukaryotic organisms and **(B)** within eukaryotic microalgae. Please note the different scale on the y-axis **(C,D)** number of papers, as reported by Web of Sciences (September 2019), containing the keywords **(C)** “metallothionein*” and **(D)** “phytochelatin*” alone or in association with the keywords “microalga*/phytoplankt*/phytobenth*.” Keyword searches were case insensitive.

Information on MTs from microbial eukaryotes different from ciliates is thus either missing or scarce (Ziller and Fraissinet-Tachet, 2018). Indeed, 5,833 eukaryotic proteins from GenBank^1^ (November 2019) are annotated as MTs, and only 153 of them are associated with microbial eukaryotes for a total of 27 genera represented (Figure 2A and Table 1). Most known MTs from microbial eukaryotes are associated with ciliates (e.g., *Tetrahymena* and *Paramecium*), Apusozoa (*Thecamonas*), as well as parasitic Apicomplexa (e.g., *Babesia, Plasmodium*, *Theileria*) or Amebae (*Entamoeba*), and six microalgal genera (*Aureococcus*, *Chlorella*, *Nannochloropsis*, *Ostreococcus*, *Symbiodinium*, and *Thalassiosira*) include marine representatives (Table 1). Although diatoms, dinoflagellates, and Haptophyta account for the vast majority of marine primary production, MTs have been predicted only in *Symbiodinium microadriaticum* (dinoflagellates; GenBank accession number OLP85454) and *T. pseudonana* (diatom; *XP_002296843*). Similarly, 137 peer-reviewed articles on microalgal MTs were published between 1995 and September 2019^2^, accounting for barely 1% of 13,761 MT-related articles (Figure 2C). Because of the high genetic diversity encompassed in eukaryotic microalgae, potentially reflecting a broad diversity of MTs, the scarcity of MT-related publications focusing on microalgae is surprising. Since MTs are highly diverse and some microalgae are known to survive in HM-contaminated environments, potentially possessing novel MT forms, future research should focus on the discovery of new MTs from microalgae following both *in silico* and experimental approaches.

**TABLE 1:** Genera of microbial eukaryotes in which metallothioneins have been predicted and eventually described^12^.

**Supergroup**	**Phylum**	**Class**	**Genus**	**No of proteins**
Alveolata	Ciliophora	Oligohymenophorea	*Tetrahymena*	65
Alveolata	Apicomplexa		*Babesia*	14
Amoebozoa	Conosa	Archamoebea	*Entamoeba*	8
Alveolata	Apicomplexa		*Plasmodium*	7
Alveolata	Apicomplexa		*Theileria*	7
Alveolata	Ciliophora	Oligohymenophorea	*Paramecium*	5
Apusozoa	Apusomonadidae	Apusomonadidae Group II	*Thecamonas*	4
Opisthokonta	Choanoflagellida	Choanoflagellatea	*Monosiga*	4
Stramenopiles	Ochrophyta	Pelagophyceae	***Aureococcus***	4
Excavata	Metamonada	Parabasalia	*Trichomonas*	3
Stramenopiles		Oomycota	*Phytophthora*	3
Alveolata	Ciliophora	Oligohymenophorea	*Ichthyophthirius*	2
Alveolata	Dinoflagellata	Dinophyceae	***Symbiodinium***	2
Amoebozoa	Evosea	Eumycetozoa	*Cavenderia*	2
Excavata	Discoba	Heterolobosea	*Naegleria*	2
Opisthokonta	Choanoflagellida	Choanoflagellatea	*Salpingoeca*	2
Opisthokonta	Mesomycetozoa	Filasterea	*Capsaspora*	2
Stramenopiles	Ochrophyta	Bacillariophyta	***Thalassiosira***	2
Stramenopiles	Oomycetes	Oomycetes	*Peronospora*	2
Alveolata	Ciliophora	Spirotrichea	*Stylonychia*	1
Amoebozoa	Conosa	Mycetozoa-Dictyostelea	*Dictyostelium*	1
Excavata	Discoba	Euglenozoa	*Trypanosoma*	1
Stramenopiles	incertae sedis	Opalinata	*Blastocystis*	1
Archaeoplastida	Mamiellophyceae	Mamiellophyceae	***Ostreococcus***	1
Archaeoplastida	Chlorophyta	Trebouxiophyceae	***Chlorella***	6
Archaeoplastida	Chlorophyta	Trebouxiophyceae	*Micractinium*	2
Stramenopiles	Ochrophyta	Eustigmatophyceae	***Nannochloropsis***	1

*^1^Data from GenBank (November 2019). ^2^Phototrophic species are in bold.*

## Glutathione and Phytochelatins in the Microalgal Realm

**FIGURE 3:**
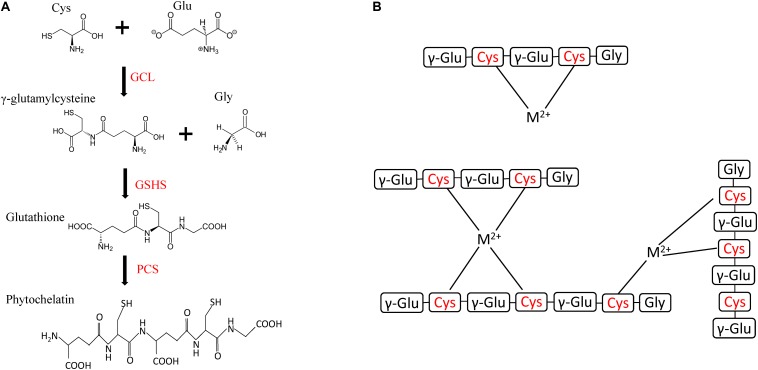
Structure and biosynthetic pathways of glutathione (GSH) and phytochelatin (PC). Three-letter abbreviations correspond to amino acid codes. **(A)** The biosynthetic pathway of GSH and PC consists of two and three reactions, respectively. First, a cysteine unit binds to the carboxylic group of the side chain of glutamic acid residue to form γ-glutamylcysteine, and this reaction is catalyzed by glutamate–cysteine ligase (GCL), then γ-glutamylcysteine binds to a glycine residue to form glutathione, and glutathione synthetase (GSHS) catalyses this reaction. PC-synthase (PCS) might then bind two or more GSH units to form phytochelatins. **(B)** Example of PC–metal complexes for divalent cations. M^2+^ denotes bivalent metal cations, whereas cysteine residues are indicated in red.

Glutathione (GSH) is a tripeptide consisting of a glutamate unit bound through the carboxyl group of its side chain to the amino group of a cysteine molecule (γ-glutamylcysteine), which is, in turn, bound to a glycine unit through a peptide bond (Figure 3A). GSH is the main redox buffer in eukaryotes and is mostly involved in the defense against oxidative (Meister and Anderson, 1983) and metal (Ahner et al., 2002) stress. *Emiliania huxleyi* cultured with up to 148 nM copper was found to release thiols, as well as GSH and cysteine, in their surrounding environment (Leal et al., 1999), suggesting a role of GSH in microalgae–metal interactions. GSH also acts as a precursor of PCs. PCs were formerly known as class III MTs and were initially identified in higher plants (Grill et al., 1985). PCs are involved in the transport of HMs from the cytosol into the vacuole in higher plants and yeasts (Cobbett and Goldsbrough, 2002). *Arabidopsis thaliana* and *Schizosaccharomyces pombe* showed an increase in sensitivity to cadmium and copper after inhibition of GSH biosynthesis (Clemens et al., 1999).

While GSH is composed by a γ-glutamylcysteine unit bound to a glycine residue, PCs consist of multiple units of γ-glutamylcysteine bound to a terminal glycine unit (Figure 3A). The typical formula of PCs is (γglu-cys)*_*n*_*-gly with 2 < *n* < 10 (Cobbett and Goldsbrough, 2002; Hirata et al., 2005). Cysteine residues present within different GSH units of a PC can form disulfide bonds and, similarly to MTs, PCs are able to chelate metallic cations through the thiol groups occurring in the side chain of the cysteine residues (Figure 3B). HM cations have been suggested to coordinate up to four sulfide groups from one or more PCs, within an HM–PC complex (Hirata et al., 2005). Apart from glycine, terminal amino acid residues, such as glutamine, glutamic acid, serine, alanine, and β-alanine, can also occur in PCs. Overall, nine different PC variants are known to date (Dennis et al., 2019).

While HMs are stably complexed by PCs in the cytosol, the lower pH present within cellular vacuoles promotes the cleavage of HM–PC complexes (Shanab et al., 2012). Greater concentrations of HMs in the vacuole, compared to the cytosol have been reported in plants (Sun et al., 2011), and HMs in the vacuoles are likely to be chelated by malic and citric acids (Haydon and Cobbett, 2007). *Chlamydomonas reinhardtii* was found to accumulate silver, mercury, and cadmium in the vacuole (Howe and Merchant, 1992), with cadmium found to be complexed by acetic and malic acids (Penen et al., 2017). High concentrations of HMs lead to an increase in vacuole size and numbers, as reported for Chlorophyta (Levy et al., 2008) and diatoms (Nassiri et al., 1997). In addition, HMs can also accumulate within other cellular organelles; *Euglena gracilis*, a vacuole-free species, typically accumulates HM complexes into chloroplasts (Mendoza-Cozatl et al., 2002) and mitochondria (Aviles et al., 2003).

Both GSH and PCs are enzymatically produced rather than genetically encoded (Figure 3A). GSH is biosynthesized after two subsequent biosynthetic reactions: the formation of γ-glutamylcysteine from cysteine and glutamic acid, catalyzed by glutamate–cysteine ligase (GCL), and the ligation of glutamylcysteine with glycine, catalyzed by the GSH synthetase (GSHS), to form GSH (Musgrave et al., 2013). GSH can eventually be bound to other γ-glutamylcysteine units for the biosynthesis of PCs (Grill et al., 1989; Cobbett and Goldsbrough, 2002), and such reaction is typically catalyzed by PC synthases (PCSs). It has been suggested that the two genes involved in GSH biosynthesis did not evolve together.

The genes coding for GCL likely arose in cyanobacteria and were then transferred to eukaryotes by horizontal gene transfer (Copley and Dhillon, 2002). In contrast to cyanobacterial GCLs, eukaryotic GCLs possess two additional cysteine residues forming a disulfide bridge playing a role against oxidative stress (Musgrave et al., 2013). Overall, GCL genes evolved into three distantly related groups, two of which are present in eukaryotes, Group II and Group III (Copley and Dhillon, 2002). GCL proteins from Group II are mostly present in heterotrophic eukaryotes, whereas GCLs from group III have a highly conserved ALXAXSPFXXGK motif (Musgrave et al., 2013) and are present in plants and microalgae. In contrast, it has been suggested that prokaryotic and eukaryotic GSHS evolved independently because of the high genetic divergence between each other (Musgrave et al., 2013).

Phytochelatins synthases were first identified and described in plants and are highly conserved in their N-terminal domain (Filiz et al., 2019). Genes coding PCSs are also present in yeasts (Clemens et al., 1999), Metazoa (Rigouin et al., 2013), and bacteria (Hirata et al., 2005). Most PCSs from plants have a plastidial origin, since they share high similarities with cyanobacterial PCSs (Hirata et al., 2005), while species adapted to low pH environments (i.e., high HM solubility), such as *Chlamydomonas acidophila* and *Dunaliella acidophila*, imported their PCS from bacteria by horizontal gene transfer (Olsson et al., 2017).

Phytochelatins and their biosynthetic enzymes are likely to be present in all microalgae; they have been reported in different species of diatoms, dinoflagellates, Haptophyta, and Chlorophyta (Ahner et al., 1995). The vast majority of genes involved in the biosynthetic pathway of PCs (GCL, GSHS, and PCS) and available on GenBank have been identified or characterized in Metazoa, fungi, and terrestrial plants; similar to MTs, less information on GCLs, GSHSs, and PCSs from microalgae is available (Figure 2A). Known microalgal genes coding GCLs, GSHSs, and PCSs have been mostly sequenced from Chlorophyta (Figure 2B). Published literature on GSH and PCs from microalgae makes also a tiny proportion of the total GSH- and PC-related studies (Figure 2D). Because of the increasing importance of phycoremediation strategies, the development of strains that can tolerate high HM concentrations is of primary importance, and biotechnological research in the coming years is likely to focus on increasing MT and PC content of microalgae. The high chemical diversity typically encompassed in microalgae suggests that some of them might contain PC variants unknown to date. Characterizing PCs and their biosynthetic genes in microalgae might lead to the discovery of new PC variants. Furthermore, knowledge of the transcription factors for these genes might help genetic engineering in developing new, HM-tolerant, strains.

The cellular levels of PCs increase at increasing HM concentrations, indicating that the biosynthesis of PCs is induced by metals (Grill et al., 1987; Ahner and Morel, 1995). Ahner and Morel (1995) observed that PC biosynthesis is taxa specific and metal specific. Although PC concentrations in *T. weissflogii* and *Tetraselmis maculata* were found to be the highest in the presence of cadmium compared to copper, zinc, and lead, *T. maculata* exhibited much higher levels of PC induction than *T. weissflogii* in cultures supplemented with zinc (Ahner and Morel, 1995). Moreover, PC concentrations in *E. huxleyi* were comparable in the presence of either cadmium or copper (Ahner and Morel, 1995), whereas Hirata et al. (2001) reported a higher PC induction with zinc compared to cadmiumin *D. tertiolecta* (Hirata et al., 2001). Since the HM concentration required to induce GCL, GSHS, and PCS varies according to the type of metal, HMs exhibiting relatively low toxicity levels can be added to culture media to enhance the cellular abundance of PCs, thus developing phenotypes more tolerant to HMs. For example, zinc addition to microalgal cultures was proven to increase tolerance toward cadmium, as found for *E. gracilis* (Sanchez-Thomas et al., 2016) and *D. tertiolecta* (Tsuji et al., 2002). The increase in PCs in the presence of HMs can also be an indirect effect. PCs have also been shown to scavenge hydrogen peroxide and superoxide radicals, suggesting to counteract oxidative stress (Tsuji et al., 2002; Hirata et al., 2005): May and Leaver (1993) demonstrated indeed an induction of GSH by hydrogen peroxide in *A. thaliana*. Yet, the biosynthesis of both GSH and PCs in *D. tertiolecta* might be induced by radical species generated under high zinc concentrations (Tsuji et al., 2002).

## Metallothioneins and Phytochelatins in Heavy Metal Phycoremediation

Because of their high genetic, functional, and chemical diversity, as well as their ability to grow and build biomass at faster rates than terrestrial plants, microalgae are receiving increasing attention for several biotechnological applications including the production of metal-binding molecules for phycoremediation purposes.

Phycoremediation consists of the removal of HMs from contaminated waters and sediments using the ability of microalgae to incorporate metal cations from their surrounding environment. In addition to their fast growth rates and their high chemical diversity, microalgae exhibit an additional advantage because of their high surface-to-volume ratio that allows HM uptake at faster rates than larger organisms. Chlorophyta, from the genera *Chlamydomonas*, *Chlorella*, and *Scenedesmus*, are mostly used for HM remediation purposes (Table 2). Since HM uptake starts with metal adsorption onto the cell wall, and such uptake is driven by the electrochemical affinity occurring between metal cations and polar groups of cell wall polymers (Kumar et al., 2015), the use of dead microalgal biomass for HM phycoremediation is also under investigation (Kumar et al., 2015). The use of dead biomass prevents the risk of ecosystem contamination if allochthonous species are used and is particularly suitable in those highly contaminated environments in which microalgal growth is inhibited (Arıca et al., 2005). However, HM uptake can be faster if living microalgae are used; in this case, passive adsorption is coupled with an active transport of HMs within the cytosol, ultimately leading to HM compartmentalization into vacuoles, chloroplasts, or mitochondria (Mendoza-Cozatl and Moreno-Sanchez, 2005; Soldo et al., 2005; Penen et al., 2017). Among the main biochemical features involved in HM uptake, the primary active transporters, metal-binding proteins, as well as enzymatically produced metal-binding peptides should be of primary interest.

**TABLE 2:** Microalgal species mostly used in heavy metal phycoremediation.

**Metal**	**Species**	**Taxon**	**Type of biomass**	**Maximum removal yield**	**References**
				**(mg g^–1^)**	
Arsenic	*Chlorella coloniales*	Chlorophyta	Live	8.97	Jaafari and Yaghmaeian, 2019
	*Scenedesmus quadricauda*	Chlorophyta	Live	89	Zhang et al., 2013
Cadmium	*Chaetoceros calcitrans*	Centric diatom	Live	1060	Sjarul and Arifin, 2012
	*Chlamydomonas reinhardtii*	Chlorophyta	Immobilized	79.7	Bayramoğlu et al., 2006
	*Chlorella sorokiniana*	Chlorophyta	Live	43	Yoshida et al., 2006
	*Chlorella sorokiniana*	Chlorophyta	Immobilized	192	Akhtar et al., 2003
	*Chlorella vulgaris*	Chlorophyta	Live	58.4	Aksu and Dönmez, 2006
	*Cladophora fracta*	Chlorophyta	Live	4.08	Lamaia et al., 2005
	*Desmodesmus pleiomorphus*	Chlorophyta	Live	85.3	Monteiro et al., 2010
	*Nannochloropsis oculata*	Eustigmatophyceae	Live	100.4	Zhou et al., 1998
	*Planothidium lanceolatum*	Pennate diatom	Live	275.51	Sbihi et al., 2014
	*Pseudochlorococcum typicum*	Chlorophyta	Live	5.48	Shanab et al., 2012
	*Scenedesmus abundans*	Chlorophyta	Live	574	Terry and Stone, 2002
	*Scenedesmus obliquus*	Chlorophyta	Live	175.6	Mehta and Gaur, 2005
	*Tetraselmis chuii*	Chlorophyta	Live	292.6	da Costa and de Franca, 1998
Chromium					
Cr^6+^	*Chlamydomonas reinhardtii*	Chlorophyta	Live	18.2	Arıca et al., 2005
	*Chlorella vulgaris*	Chlorophyta	Live	33.8	Dönmez et al., 1999; Aksu and Dönmez, 2006
	*Dunaliella* sp.1	Chlorophyta	Live	58.3	Dönmez and Aksu, 2002
	*Dunaliella* sp.2	Chlorophyta	Live	45.5	Dönmez and Aksu, 2002
	*Scenedesmus incrassatulus*	Chlorophyta	Live	4.4	Jácome-Pilco et al., 2009
Cr^3+^	*Chlorella sorokiniana*	Chlorophyta	Immobilized	69.26	Akhtar et al., 2008
	*Chlorella miniata*	Chlorophyta	Live	41.12	Han et al., 2006
	*Oedogonium* sp.	Chlorophyta	Live	75	Bakatula et al., 2014
Cobalt	*Chlorella coloniales*	Chlorophyta	Live	9.24	Jaafari and Yaghmaeian, 2019
	*Oedogonium* sp.	Chlorophyta	Live	70	Bakatula et al., 2014
Copper	*Asterionella formosa*	Pennate diatom	Live	1.1	Tien et al., 2005
	*Aulacoseira varians*	Centric diatom	Live	2.29	Tien et al., 2005
	*Chlamydomonas reinhardtii*	Chlorophyta	Live	6.42	Macfie and Welbourn, 2000
	*Chlorella fusca*	Chlorophyta	Live	3.2	Dönmez et al., 1999
	*Chlorella pyrenoidosa*	Chlorophyta		2.4	Yan and Pan, 2002
	*Chlorella sorokiniana*	Chlorophyta	Live	46.4	Yoshida et al., 2006
	*Chlorella* spp.	Chlorophyta	Live	220	Doshi et al., 2006
	*Chlorella vulgaris*	Chlorophyta	Free	76.71	Mehta and Gaur, 2001
	*Desmodesmus* sp.	Chlorophyta	Live	33.4	Rugnini et al., 2017
	*Planothidium lanceolatum*	Pennate diatom	Live	134.32	Sbihi et al., 2014
	*Scenedesmus obliquus*	Chlorophyta	Live	1.8	Yan and Pan, 2002
	*Oedogonium* sp.	Chlorophyta	Live	75	Bakatula et al., 2014
Lead	*Chlamydomonas reinhardtii*	Chlorophyta	immobilized	380.7	Bayramoğlu et al., 2006
	*Chlorella vulgaris*	Chlorophyta	Live	17.2	Sandau et al., 1996
	*Oedogonium* sp.	Chlorophyta	dried	145	Gupta and Rastogi, 2008
	*Pseudochlorococcum typicum*	Chlorophyta	Live	4.49	Shanab et al., 2012
Mercury	*Chlamydomonas reinhardtii*	Chlorophyta	immobilized	106.6	Bayramoğlu et al., 2006
	*Oedogonium* sp.	Chlorophyta	Live	60	Bakatula et al., 2014
	*Pseudochlorococcum typicum*	Chlorophyta	Live	15.13	Shanab et al., 2012
Nickel	*Chlorella miniata*	Chlorophyta	Live	1.37	Wong et al., 2000
	*Chlorella* spp.	Chlorophyta	Live	122	Doshi et al., 2006
	*Chlorella* spp.	Chlorophyta	Immobilized	28.5	Maznah et al., 2012
	*Chlorella vulgaris*	Chlorophyta	Live	15.4	Al-Rub et al., 2004
	*Chlorella miniata*	Chlorophyta	Live	0.6	Wong et al., 2000
	*Oedogonium* sp.	Chlorophyta	Live	65	Bakatula et al., 2014
Zinc					
	*Chlorella vulgaris*	Chlorophyta	Live	9.3	Rugnini et al., 2017
	*Chlorella sorokiniana*	Chlorophyta	Live	42	Yoshida et al., 2006
	*Planothidium lanceolatum*	Pennate diatom	Live	118.66	Sbihi et al., 2014
	*Scenedesmus obliquus*	Chlorophyta	Live	836.5	Monteiro et al., 2011
	*Scenedesmus subspicatus*	Chlorophyta	Live	72.06	Schmitt et al., 2001
	*Oedogonium* sp.	Chlorophyta	Live	70	Bakatula et al., 2014

The intracellular transport of HMs and their accumulation within specific organelles is thus controlled by MTs, PCs, as well as other HM-binding molecules, such as polyphosphate bodies. While HM biosorption rates can be improved by manipulating the physico-chemical conditions (temperature, pH) to which both HMs and microalgal substrates are exposed, in order to enhance HM bioaccumulation, researchers have recombinantly expressed import–storage systems, which include channels, secondary carriers, as well as primary active transporters (Diep et al., 2018). Although most of the genetic manipulations aimed to improve metal uptake have been carried out on bacteria (Deng and Jia, 2011), yeasts (Shen et al., 2012), or higher plants (Ueno et al., 2011), a few studies focused on microalgae (Cai et al., 1999; Ibuot et al., 2017). HM-phycoremediation can be improved, in the next decade, by searching new metal-binding peptides naturally occurring in microalgae, as well as by applying genetic engineering techniques aimed at developing mutants able to tolerate high HM concentrations and to rapidly uptake and immobilize such metals.

## Metallothioneins and Phytochelatins: Other Biotechnological Applications

Since MTs increase at increasing HM concentrations, biotechnological research for biomonitoring applications also focuses on MTs for developing “biosensors” to detect HM pollution. Most of these applications are based on the immobilization of MTs onto metal electrodes. HM–MT interactions are then recorded based on changes in capacitance (Corbisier et al., 1999) or potential (Adam et al., 2005). Alternatively, whole-cell based biosensors have been also developed. MTs from the ciliate *Tetrahymena thermophila* were fused with luciferase gene to develop *T. thermophila* mutants, and luciferase activity was measured in the presence of cadmium, copper, zinc, lead, arsenic, and mercury exhibiting correlation to HM concentration (Amaro et al., 2011). Overall eukaryote-based biosensors for HM detection have been developed for yeasts, microalgae, and ciliates, with the latter preferred because of the lack of cell wall in the vegetative phase of ciliates (Gutiérrez et al., 2015). HMs can indeed rapidly penetrate the cytosol of cell wall-free species, subsequently interacting with modified MTs. Cell wall-free stages are thus more suitable for the development of biosensors, including MT-based biosensors. Cell wall-free mutants of *C. reinhardtii* possessing MTs fused with yellow fluorescent proteins, have been used to monitor the intracellular accumulation of mercury, cadmium, lead, zinc, and copper (Rajamani et al., 2014).

In addition to biosensors and bioremediation, the use of MTs and PCs for metal transport can also have some applications in Biomedical Sciences. In general, MTs or PCs might be used either to supply essential metals to humans or animals, or to remove toxic HMs from contaminated tissues of living organisms. The strong affinity of MTs and PCs toward HMs, including essential metals, such as copper and zinc, suggests indeed their potential use as drug transporters in patients affected by anemia or other blood diseases, in order to increase absorption of essential metals introduced by the diet.

In the human body, various isoforms of MTs are expressed in a tissue-specific pattern and may play distinct roles in the different cell types (Wunderlich et al., 2010; Summermatter et al., 2017). The distribution of the different MT isoforms in tissues might be linked to a disease. For example, an increase in gene expression of certain MT isoforms has been reported for breast, renal, bladder, prostate, and thyroid cancer (Chung et al., 2008; Thirumoorthy et al., 2011; Gumulec et al., 2014), whereas MTs were found to be downregulated in patients affected by liver cancer (Thirumoorthy et al., 2011), indicating that the expression of MTs is cancer specific rather than universal to all human tumors. This suggests that tumor prognosis might be either directly or indirectly linked with both gene expression and isoform type of MTs. Because of this relationship between tumors and MT expression, biomedical research might focus on developing MT-based biomarkers and diagnostic tools for cancer prognosis.

Furthermore, since MTs can scavenge radical species exhibiting some antioxidant properties, cosmeceutical and nutraceutical application of microalgae enriched in MTs are also under investigation. Antioxidant properties of MTs are due to their high cysteine content that induces the formation of metal–thiolate clusters (Carpenè et al., 2007). Zhang et al. (2006) evaluated the impact of UV-B on *C. reinhardtii* and found an increased survival rate for mutants engineered with the insertion of a human MT gene compared to the wild type. MTs have been suggested to regulate cell growth and protecting the body against oxidative stress (Kumari et al., 1998) and to mediate zinc homeostasis (Krezel and Maret, 2017), being directly involved in immune system functionality.

## Current Analytical Techniques for the Determination of MTs and PCs

Despite the attention toward MTs for biotechnological purposes, the chemical and molecular determination of MTs is yet to be fully revised. The main chemical methods applied to date are mostly based on spectrophotometry and chromatography. A simple technique for MT determination is based on the quantification of thiol-rich compounds, as described by Ellman (1959). This method has been successfully applied to investigate cadmium tolerance in *P. tricornutum* (Torres et al., 1997) and *Dunaliella salina* (Folgar et al., 2009). This technique is thus based on the quantification of sulfide groups, and GSH is usually used for calibration purposes.

Metallothionein structure can be determined using ^113^Cd- and ^1^H- nuclear magnetic resonance (Duncan et al., 2008), electron paramagnetic resonance (Krepkiy et al., 2003), Raman spectroscopy (Giner et al., 2018), circular dichroism (Yang et al., 2019), Mossbauer spectroscopy (Afonso et al., 2004), and X-ray absorption near-edge structure spectroscopy (Manceau et al., 2019). Mass spectrometry (MS) can be coupled with electrospray ionization (ESI) or matrix-assisted laser desorption/ionization (MALDI) (Ryvolova et al., 2011). MS-ESI has the advantage to preserve the integrity of the molecular ions and is very effective for a precise determination of the molecular weight of the peptides, whereas MS-MALDI is typically coupled with time-of-flight instruments for unambiguous identification of the primary structure of proteins (Liu and Schey, 2005).

Inductively coupled plasma-mass spectrometry (ICP-MS) allows measuring the metal content of proteins even at very low concentrations (Ryvolova et al., 2011; Hauser-Davis et al., 2012). Chromatographic methods for MT determination include size exclusion chromatography (Wolf et al., 2009), ion exchange (Infante et al., 2006), and two-dimensional high-performance liquid chromatography (HPLC) coupled with ICP-MS (Miyayama et al., 2007). Resonance light scattering (RLS) was applied using Eosin Y as a dye (Xiao et al., 2015) or phenantroline-Cu chelate as a probe (Xiao et al., 2014) and revealed a cost effective and rapid technique for the determination of MTs at nanomolar levels.

Metallothioneins can also be determined using immunoassays, Western blot, and mRNA sequencing. The enzyme-linked immunosorbent assays (ELISA) is the most common immunochemical method to detect MTs, and is used in both humans and experimental animals (Saito et al., 2013; Nakazato et al., 2014). Sequencing of mRNA has been used to determine the basal expression (Kim et al., 2002) of MTs or variations in their expression levels after exposure to metals (Wu et al., 2008) or for medical purposes (Nagamine et al., 2005). Western blot and mRNA sequencing were also applied for pharmacological (Grzegrzolka et al., 2019) or cytotoxic assays (Li et al., 2019).

Similarly to MTs, PC concentration in cells can also be measured using HPLC-based techniques. The molecular mass of PCs can be predicted from the number of GSH monomers, as well as the presence and the nature of bound metals. A database that includes the molecular masses of all detected and predicted PCs in plants has been recently published (Dennis et al., 2019). Since both MTs and PCs contain cysteine residues, many analytical techniques used for the determination of MTs can also be applied to detect and quantify PCs within biomass. GSH and PCs are typically quantified using HPLC based on postcolumn reaction with Ellman’s reagent (Tukendorf and Rauser, 1990). This technique was applied to quantify the PC content in *Chlorella vulgaris* (Bajguz, 2011). The concentrations of cysteine, GSH, and PCs can also be determined simultaneously using HPLC with electrochemical detection (Potesil et al., 2005) or gradient elution (Wu et al., 2016).

## Challenges for Genetic Engineering

Genetic engineering techniques aimed at increasing the cellular concentrations of metal-binding proteins have been mostly applied to bacteria, yeasts, and terrestrial plants. Cellular uptake of HMs from the surrounding environment can be improved by increasing the cellular concentrations of primary active transporters. Modified bacteria (*Lactobacillus plantarum*) and plants (*T. caerulescens*) exhibiting increased cellular proportions of primary active transporters were indeed found to uptake larger amounts of cadmium compared to the wild types (Deng et al., 2007; Ueno et al., 2011). Similarly, increased copper uptake rates were found in engineered *Enterobacter hirae* (Zagorski and Wilson, 2004). Since polyphosphate bodies are also known to bind metals (Wang and Dei, 2006), overexpression of polyphosphate kinases has been applied to improve mercury uptake (Kiyono et al., 2003).

The microalgal species most widely used for genetic engineering is the model Chlorophyta *C. reinhardtii* (Leon-Banares et al., 2004). The first application of genetic engineering aimed at improving HM uptake dates back to 20 years and focused on the expression of exogenous MTs in *C. reinhardtii* (Cai et al., 1999); the resulting transgenic strain exhibited a higher growth rate in the presence of 40 μM cadmium, compared to the wild type. MT genes from *Festuca rubra* (higher plant) were cloned into the plastidial genome of *C. reinhardtii* resulting in a mutant capable of growing in the presence of cadmium concentrations as high as 80 μM (Han et al., 2008).

However, it has been shown that overexpression of MTs may not be sufficient to improve the metal uptake, since high abundances of these proteins may trigger the aggregation of misfolded forms, leading to the formation of inefficient or unstable proteins and resulting in lower metal uptake rates (Diep et al., 2018). In contrast, the overexpression of MT fusion proteins, resulting from the combination of MTs and soluble proteins, can lead to increased metal uptake rates. Chicken MTs were fused with yellow fluorescent proteins and expressed in *C. reinhardtii* to monitor the intracellular accumulation of different metals (Rajamani et al., 2014); the engineered *C. reinhardtii* revealed highly efficient as a biosensor for mercury, cadmium, lead, and, to a lesser extent, zinc and copper.

Other applications of MT fusion proteins were carried out using bacteria, yeasts, or plants as expression hosts. For example, the fusion of MT genes from *Corynebacterium glutamicum* with histidine-tagged proteins, and the cloning of the resulting MT fusion protein into *Escherichia coli*, led to increased accumulation of lead, zinc, and cadmium compared to the wild type (Jafarian and Ghaffari, 2017). Two common MT-based fusion proteins, used in genetic engineering to improve metal bioaccumulation, result from the fusion of MTs with either green fluorescence proteins or glutathione-S-transferase (GST) (Diep et al., 2018). Ruta et al. (2017) fused 11 plant MTs with myristoylated green fluorescent proteins in order to target the fusion product toward the inner face of the cell membrane in *Saccharomyces cerevisiae.* Recombinant yeasts were then found to accumulate greater amounts of cobalt, copper, zinc, and cadmium compared to the wild type (Ruta et al., 2017). Green fluorescent proteins have also been fused with human MTs, and the resulting fusion gene cloned in *S. cerevisiae* led to an increase in copper accumulation (Geva et al., 2016). MTs from *Oryza sativa* were fused with GST, and they were expressed in *E. coli*; the recombinant bacteria were found to accumulate greater amounts of mercury (Shahpiri and Mohammadzadeh, 2018). GST-MTs developed from *Pisum sativum* were cloned into the photosynthetic bacterium *Rhodopseudomonas palustris*, and the recombinant *R. palustris* was found to be more tolerant to mercury (Deng and Jia, 2011). Kim et al. (2005) cloned GST-MT into *E. coli* and showed increased uptake of cadmium from the recombinant bacterium compared to the wild type. *E. coli* mutants containing human MT also exhibited higher cadmium and arsenic uptakes compared to the wild type (Jahnke et al., 2011). Human MTs have also been cloned into the genome of *Mesorhizobium huakuii*, a bacterial symbiont of leguminous roots, to improve cadmium uptake (Sriprang et al., 2002). Ruiz et al. (2011) cloned MTs from a mouse into *E. coli* for mercury remediation purposes. The HM uptake rates of engineered strains might also vary according to the intracellular localization of MTs. For example, engineered *E. coli* with MTs expressed in the periplasmic compartment exhibited a higher cadmium biosorption efficiency compared to the recombinant strains, in which MTs had been targeted toward the cytosol (Kao et al., 2006). In contrast, Sauge-Merle et al. (2012) found greater bioaccumulation rates in *E. coli* overexpressing MT fusion proteins in the periplasm only for copper, whereas cadmium, arsenic, mercury, and zinc were better adsorbed by recombinant *E. coli* with MT fusion proteins targeted toward the cytosol. A recent study expressed MT fusion proteins in *E. coli* and extracted the resulting proteins to make a biosorbent used for the removal of lead and zinc (Mwandira et al., 2020).

Genetic engineering also focused on enzymatically produced peptides, such as PCs. Overexpression of GCL, GSHS, and PCS enzymes has also been applied to increase the cellular concentration of PCs (Diep et al., 2018). For example, Olsson et al. (2017) increased cadmium tolerance in *E. coli* by cloning and expressing a PCS from *C. acidophila*. However, the intracellular environment is typically more reduced than the outer environment in both prokaryotic (Bessette et al., 1999) and eukaryotic (Go and Jones, 2008) cells, thus affecting the formation of disulfide bridges within MTs. Also, the overexpression of both MTs and PCs in cells increases the demand for cysteine biosynthesis negatively impacting the growth.

Genetic engineering techniques thus contributed in developing microbial mutants capable of greater HM uptake from the environment, especially through the enhanced expression of MTs. However, despite the great potential of eukaryotic microalgae for HM phycoremediation, the number of microalgal mutant developed for this purpose is still limited. This is likely due to the difficulties in genetically modifying eukaryotic cells compared to prokaryotes, and to the little information available on MTs from microbial eukaryotes. Future progresses in genetic engineering should facilitate the development of eukaryotic mutants. Sequencing new genomes and metagenomes from polluted environments and bioinformatic mining of pre-existing databases might help in finding novel microalgal MTs.

## Author Contributions

AS gave her contribution on analytical techniques for the determination of metallothioneins and phytochelatins. AS and TC investigated current strategies for heavy metal phycoremediation. MB reviewed genetic engineering techniques to improve heavy metal uptake from microalgae. FD and CS highlighted potential applications of metallothioneins in pharmaceutics and nutraceutics as well as in biomedical sciences. SB and CB conceived the manuscript, which was drafted by SB and critically reviewed and approved by all the co-authors.

## Conflict of Interest

The authors declare that the research was conducted in the absence of any commercial or financial relationships that could be construed as a potential conflict of interest.

## Footnotes

1 www.ncbi.nlm.nih.gov/

2 www.webofknowledge.com
